# Retraction: miR-362-3p functions as a tumor suppressor through targeting MCM5 in cervical adenocarcinoma

**DOI:** 10.1042/BSR-2018-0668_RET

**Published:** 2024-11-06

**Keywords:** biomarker, cervical cancer, MCM5, microRNA, miR-362-3p

This article “MiR-362-3p functions as a tumor suppressor through targeting MCM5 in cervical adenocarcinoma” DOI: 10.1042/BSR20180668 is being retracted from *Bioscience Reports* at the request of the Editor-in-Chief and the Editorial Board. This follows receipt of a notification from a reader, alerting the Editorial Board to Figure 2E Hela β-actin panel, which seems to appear as Figure 4 A-172 β-actin panel in a subsequent paper by Zhao et al, 2019 (DOI: 10.1002/jcb.28430) [retracted] with contrast alteration. Upon review of the case, the Editorial Office identified further links to papers by different authors, and suspect the peer review process was likely compromised for this article. Therefore the Editorial Board no longer considers the findings reliable, and stands by the decision to retract. The authors have been contacted with regards to the retraction and have not responded to the Journal's queries or the concerns raised.

